# Acknowledgment to Reviewers of *Dentistry Journal* in 2020

**DOI:** 10.3390/dj9020015

**Published:** 2021-01-28

**Authors:** Dentistry Journal Editorial Office

**Affiliations:** MDPI AG, St. Alban-Anlage 66, 4052 Basel, Switzerland

Peer Peer review is the driving force of journal development, and reviewers are gatekeepers who ensure that *Dentistry Journal* maintains its standards for the high quality of its published papers. Thanks to the cooperation of our reviewers, in 2020, the median time to first decision was 20 days and the median time to publication was 49 days. The editors would like to express their sincere gratitude to the following reviewers for their precious time and dedication, regardless of whether the papers were finally published:
Aartman, Irene Helena AdrianaLiu, ZhongweiAbuzar, MenakaLlena, Carmen C.Adekoya, Timothy O.Lo Giudice, AntoninoAdrover, AlessandraLo Giudice, GiuseppeAfrashtehfar, Kelvin IanLoewy, ZviAgrawal, PriyankaLombardi, TommasoAgustín Panadero, RubénLončar Brzak, BožanaAl-Ezzi, MinanLopez-Jornet, PiaAli, AimanLops, DiegoAlikhani, ManiLuong, MinhAl-Rudainy, DhelalLupi, Saturnino MarcoAl-Saleh, Mohammed A.Q.Machado, VanessaAlstergren, PerMačiulskienė, VitaAna Maria Cristina, TancuMagan-Fernandez, AntonioAnastasiou, AntoniosMakvandi, PooyanAndabak Rogulj, AnaManakil, JaneAnderson, AlexaMariggiò, Maria A.Andreana, SebastianoMarshman, ZoeAndrews, JeanMartinez, YolandaAndrukhov, OlehMartinotti, SimonaAoki, AkiraMartins, FranciscoArjunan, PachiappanMascitti, MarcoArkadiusz, DziedzicMaspero, CinziaArnabat-Domínguez, JosepMatarese, GiovanniAsa’ad, FarahMatys, JacekAta-Ali, JavierMayr, AstridAtsawasuwan, PhimonMeghil, MohamedAttia, SamehMeister, JörgBaghdadi, Ziad D.Melo, MaríaBakarčić, DankoMenchini Fabris, Giovanni BattistaBallini, AndreaMendes, JoaquimBanas, JeffreyMendes, José ManuelBani, DanieleMertens, ChristianBardag-Gorce, FawziaMesquita-Guimarães, JoanaBasic, KresimirMester, AlexandruBatchelor, PaulMeštrović, SenkaBechir, AnamariaMetere, AlessioBecker, KathrinMeyer, Beau D.Begnoni, GiacomoMeyer-Lueckel, HendrikBel’skaya, LyudmilaMichl, ThomasBencharit, SompopMihali, SorinBennardo, FrancescoMihelogiannakis, DimitriosBenz, KorbinianMikrogeorgis, GeorgiosBerg, Britt-IsabelleMilleman, Jeffery L.Bernardi, SaraMiranda-Rius, JaumeBerton, FedericoMirmohammadi, Hesam H.Bianchi, JonasMishu, MasumaBirjandi, Anahid AhmadiMitsea, Anastasia G.Bjerklin, KristerMoharamzadeh, KeyvanBordea, Ioana RoxanaMontaruli, GrazianoBorzabadi-Farahani, AliMoore, RodBotzenhart, UteMourão, Carlos FernandoBourgeois, DenisMudde, AartBragazzi, Nicola LuigiMuntean, AlexandrinaBrailo, VlahoMunz, MatthiasBright, RichardMuruganandan, ShanmugamBrkić, HrvojeMussano, FedericoBrucoli, MatteoMyers, VickiBruno, VincenzoMyllymaa, SamiBurgess, JacquelineNakamura, NaokoBurkhard, John PatrikNalliah, RomeshCalarco, AnnaNascimento, Gustavo GCalvo, Jean MarieNeumann, Ana SolariCampisi, GiuseppinaNguyen, Vu-HieuCampus, GuglielmoNicolas-Silvente, Ana IsabelCapocasale, GiorgiaNicolescu, Mihnea-IoanCarpegna, GiorgiaNocca, GiuseppinaCarrilho, EuniceNoren, HasmunCarrouel, FlorenceNosotti, Maria GiuliaCasanas, ElisabethNota, AlessandroCenariu, MihaiNowicki, MarekCha, SeungheeNucera, RiccardoChang, Hsueh-WeiOgórek, RafałChang, Yu-ChaoOho, TakahikoChaushu, GavrielOnisor, FlorinChavis, Sydnee EOpydo-Szymaczek, JustynaChen, Chia-YuOteri, GiacomoChen, LinOttolenghi, LiviaChenchev, IvanOuchi, TakehitoCheon, KyoungaOvsenik, MajaChou, Tzu-ChiehPaap, Kenneth R.Chrcanovic, BrunoPagano, StefanoCicciù, MarcoPałka, KrzysztofClaiborne, DenisePalma, Paulo JorgeCoelho, AnaPalmer, Robert J.Collart-Dutilleul, Pierre-YvesPalomo, LeenaColombo, JohnPan, HongyingConrads, GeorgPandey, Ravindra K.Contaldo, MariaPaolino, GiovanniCorridore, DenisePatini, RomeoCosma, PinalysaPatterson, JenniferCristache, Corina MarilenaPawlaczyk-Kamieńska, TamaraCurtis, DenicePelivan, IvicaCurto, AdrianPenlington, ChrisCutler, Christopher WPerez-Sayans, MarioD’Addazio, GianmariaPeroš, KristinaDalessandri, DomenicoPfeiffer, SusanD’anto, VincenzoPielech, MelissaDarvell, Brian WPippi, RobertoDavidovich, EstiPirani, ChiaraDawes, JudithPizzi, SilviaDe Biase, AlbertoPodgórski, MaciejDe Stefani, AlbertoPolk, Deborah EDel Val, JesúsPommer, BernhardDel Vecchio, AlessandroPorojan, LilianaDenis, FredericPortelli, MarcoDi Blasio, AlbertoPou, JuanDi Fiore, AdolfoProença, LuísDi Gianfilippo, RiccardoPurdoiu, Robert CristianDi Stasio, DarioQuang Le, BachDiachkova, EkaterinaQuinzi, VincenzoDiogo, PatríciaRai, Nayanjot KDioguardi, MarioRanzato, EliaDoerrler, WilliamRapone, BiagioDos Santos, João Miguel MarquesRe, DinoDrukteinis, SauliusRechmann, PeterDudkin, Semyon V.Renapurkar, ShravanDumancic, JelenaRequicha, JoãoDussault, PatrickRiad, AbanoubDyszkiewicz-Konwińska, MartaRibeiro, ApoenaDziedzic, ArkadiuszRíos-Carrasco, BlancaEgloff-Juras, ClaireRizvi, Syed A. A.Ekambaram, ManikandanRobb, Nigel DEl Mobadder, MarwanRoberts, Michael WEl-Bialy, TarekRoccuzzo, AndreaEl-Naaj, Imad AbuRodríguez-Archilla, AlbertoEmmanouil, DimitrisRodríguez-Lozano, Francisco JavierEnrico, Gherlone FeliceRomano, FedericaEramo, StefanoRubilotta, EmanueleFarahani, AliRunte, ChristophFarronato, DavideRusu, Laura CristinaFarronato, GiampietroSaarela, RiittaFaus-Matoses, VicenteSabol, IvanFawzy, AmrSabounchi, Sepideh SeyedzadehFelice, RobertoSachelarie, LilianaFelix Gomez, Grace GomezSaito, AtsushiFeraru, Ion VictorSakata, JunkiFernandes Fagundes, NathaliaSakkas, Giorgos K.Ferrarotti, FrancescoSantacroce, LuigiFestila, Dana GabrielaSarafianou, AspasiaField, JamesSardana, DiveshFigueiral, Maria HelenaSato, TakuichiFigueras-Alvarez, OscarSatou, RyouichiFine, PeterSayardoust, SharielFiorillo, LucaScambler, SashaFlaherty, KevinScardina, Giuseppe AlessandroForner, LeopoldoScheau, CristianFortunato, LeonzioSchmidt, JanGabrić, DraganaSchmitz, IngeGallob, John T.Schmitz, Johannes H.Galluccio, GabriellaScholkmann, FelixGandedkar, NarayanScoffield, JessicaGaraicoa-Pazmino, CarlosŠegović, SanjaGarcía-Pola, María JoséSeok, HyunGeorge, RoySerpico, RosarioGerreth, KarolinaSetty, BhuvanaGhassib, IyaSfeatcu, Ionela RuxandraGhislanzoni, Luis HuancaShahmoradi, MahdiGinszt, MichałSharma, DileepGiudice, AmerigoShiau, Harlan J.Giudice, Giuseppe LoShimpi, NeelGlavina, DomagojShopova, DobromiraGómez-Salgado, JuanSimancas-Pallares, MiguelGonzales, Cara B.Simione, MegGorseta, KristinaSimonetta, D’ErcoleGoto, TakaharuSinelnikov, MikhailGradova, MargaretSinjari, BrunaGrassia, VincenzoSivolella, StefanoGrimm, Wolf-DieterSkapetis, TonyGrippaudo, CristinaSkrinjar, IvanaGrzech-Leśniak, KingaSkrinjaric, TomislavGu, ChunjuSmailienė, DaliaGurzawska, KatarzynaSokal-Gutierrez, KarenGussy, MarkSon, KeunBaDaGutierrez-Perez, Jose-LuisSong, Linyong (Leon)Ha, William N.Song, Min-juHagedorn, LindaSpagnuolo, GianricoHagenfeld, DanielSpendier, KathrinHanisch, MarcelSry, VanneiHaraszthy, VioletStaderini, EdoardoHattab, Faiez N.Stocchero, MicheleHayashi, Jun-ichiroStoichkov, BiserHeaton, LisaSu, Kuo-ChihHector, MarkSuga, TakayukiHegedűs, CsabaSugiyama, MasaruHenningsen, AndersSukegawa, ShintaroHeumann, ChristianSuzuki, MaikoHeyman, Richard ESzekely, MelindaHirata, RonaldoSzkaradkiewicz, AndrzejHoefert, SebastianTallarico, MarcoHöhne, ChristianTanaka, TakujiHong, Jiann-RueyTartaglia, GianlucaHoover, JayTavelli, LorenzoHorowitz, AliceTaylor, GreigHorwitz, JacobTenore, GianlucaHowe, BrianTeoh, Yu-YaoHsu, Jui-TingTestarelli, LucaHuck, OlivierThomas, Tami L.Hughes, Christopher V.Tirino, VirginiaIbrahim, MohamedToffoli, AndreaIldikó, TarTomasic, IvanIliadi, AnnaTomofuji, TakaakiImai, TomoakiTosios, KonstantinosImbery, Terence A.Trajkovski, BrankoImre, Marina MelescanuTriplett, R. GilbertInchingolo, FrancescoTsolakis, Apostolos I.Isola, GaetanoTsuchida, SachioIto, KanadeTurner, JonathanIvanišević, AnaUpadhyaya, JasbirIwata, TakanoriUpshur, Carole CJaeger, AndreasValera-Gran, DesiréeJaradat, NidalVan Den Bergh, HubertJentschke, MatthiasVardas, EmmanouilJokić, Nataša IvančićVasconcelos, Mário RJost-Brinkmann, Paul-GeorgVelliyagounder, KabilanJurado, CarlosVerket, AndersKaczor-Urnanowicz, Karolina ElzbietaVerzak, ŽeljkoKarolewicz, BożenaVieyra, Jorge ParedesKarpiński, Tomasz M.VIZUREANU, PetricaKau, Chung HowVouros, IoannisKhaled, AhmedVozza, IoleKim, Jin-BomWang, ChangxiangKim, Seong-GonWang, Chih-hungKim, Sin-youngWang, HaoyuKim, SooyeonWang, Peng-HuiKim, SunilWang, QinyiKinane, Denis FWashio, AyakoKingsley, KarlWertz, Philip W.Knaup-Gregori, PetraWolf, KatarinaKobayashi, JunWu, Aaron Yu-JenKobayashi, YoshikazuYagami, KimitoshiKoizumi, HiroyasuYamaguchi, SatoshiKorsunsky, Alexander M.Yang, ShanKorupalli, ChiranjeeviYassen, GhaethKoyama, ShihokoYeo, In-SungKrishnan, HariniYepes, Juan F.Kulkarni, GajananYeung, Andy Wai KanKum, Kee-YeonYi, Sun K.Kusnoto, BudiYu, NingKwon, Jae SungYu, QingsongLach, SławomirYu, Shan-HueyLagravère, Manuel O.Yuan, KuoLajolo, CarloYuan, SiyangLam, OttoZandinejad, AmiraliLan, Ting-HsunZara, FrancescaŁapińska, BarbaraZaric, SvetislavLazăr, LuminițaZhan, LingLEBON, NicolasZhang, AnqiLee, Ji HyeZhou, ChengjiLevartovsky, ShifraZieliński, RafałLi, PingZlatarić, Dubravka KnezovićLim, Lum PengZotti, FrancescaLin, Wei-ChunZubizarreta-Macho, Álvaro

